# Diagnostic Performance of Afirma and Interpace Diagnostics Genetic Testing in Indeterminate Thyroid Nodules: A Single Center Study †

**DOI:** 10.3390/cancers15072098

**Published:** 2023-03-31

**Authors:** Emad Kandil, Tyler A. Metz, Peter P. Issa, Mohamed Aboueisha, Mahmoud Omar, Abdallah S. Attia, Bert Chabot, Mohammad Hussein, Krzysztof Moroz, Mohamed Shama, Eman Toraih

**Affiliations:** 1 Department of Surgery, Tulane University School of Medicine, New Orleans, LA 70112, USA; 2 Tulane University School of Medicine, New Orleans, LA 70112, USA; 3 School of Medicine, Louisiana State University Health Sciences Center, New Orleans, LA 70112, USA; 4 Department of Otolaryngology-Head and Neck Surgery, Faculty of Medicine, Suez Canal University, Ismailia 41522, Egypt; 5 Department of Pathology, Tulane University School of Medicine, New Orleans, LA 70112, USA; 6 Genetics Unit, Department of Histology and Cell Biology, Faculty of Medicine, Suez Canal University, Ismailia 41522, Egypt

**Keywords:** indeterminate thyroid nodules, Afirma, Interpace Diagnostic, statistical measures, genetic testing

## Abstract

**Simple Summary:**

Molecular testing can stratify the risk of malignancy among indeterminate thyroid nodules and subsequently reduce the need for diagnostic surgery. We assessed the performance of the GSC + XA; ThyGeNEXT + ThyraMIR, and GSC + GEC molecular platforms to elucidate their diagnostic accuracy. All three platforms were reliable for ruling out thyroid cancer, with a tendency for the newest Afirma genetic testing platform (GSC + XA) to outperform the other platforms.

**Abstract:**

Indeterminate thyroid nodules (ITN) represent 20–30% of biopsied nodules, with a 10–60% risk of malignancy. Molecular testing can stratify the risk of malignancy among ITNs, and subsequently reduce the need for unnecessary diagnostic surgery. We aimed to assess the performance of these molecular tests at a single institution. Patients with Bethesda III, IV, and V nodules with Afirma and Interpace Diagnostics genetic testing data from November 2013 to November 2021 were included. Three cohorts were formed, including GSC + XA, ThyGeNEXT + ThyraMIR, and GSC + GEC. Statistical analysis determined the sensitivity, specificity, positive predictive value (PPV), negative predictive value (NPV), diagnostic odds ratio (DOR), and accuracy of each type of testing. The PPV of nodules undergoing genetic testing by ThyGeNEXT + ThyraMIR (45.00%, 95%CI: 28.28–62.93%, *p* = 0.032) and GSC + XA (57.14%, 95%CI: 29.32–81.08%, *p* < 0.001) were superior to that of GEC + GSC (30.72%, 95%CI: 26.83–34.90%). The NPV was above 85% in all cohorts, suggesting overall suitable rule-out tests. The Afirma platform (GSC + XA) had the highest NPV at 96.97%. The overall accuracy for nodules undergoing ThyGeNEXT + ThyraMIR was 81.42% (95%CI: 73.01–88.11%, *p* < 0.001). A total of 230 patients underwent thyroidectomy, including less than 60% of each of the ThyGeNEXT + ThyraMIR and GSC + XA cohorts. Specifically, only 25% of patients in the GSC + XA cohort underwent surgery, considerably decreasing the rate of unnecessary surgical intervention. Sub-group analysis, including only patients with surgical pathology, found that PPV tended to be higher in the GSC + XA cohort, at 66.67% (95%CI: 37.28–87.06%), as compared to the ThyGeNEXT + ThyraMIR cohort, at 52.94% (95%CI: 35.25–69.92%). The Afirma genetic testing platform GSC + XA outperformed the other platforms with regards to both PPV and NPV and decreased the rate of surgery in patients with ITNs by 75%, significantly preventing unnecessary surgical intervention.

## 1. Introduction

Thyroid nodules are common, prevalent in up to two-thirds of the general population [1]. While most thyroid nodules are benign, they can represent concerning pathology which may significantly adversely impact patient survival. As a result, the accurate assessment of thyroid nodules is important.

Thyroid nodule assessment typically includes sampling by fine-needle aspiration (FNA). Current (2015) American Thyroid Association (ATA) guidelines recommend cytopathological evaluation with FNA biopsy of thyroid nodules that meet specific criteria, such as solid nodules greater than 1 cm, complex nodules greater than 1.5 cm, and thyroid nodules with suspicious features of malignancy on ultrasound [2]. FNA is a safe and minimally-invasive procedure, typically performed under sonographic guidance, allowing for cytopathological assessment of the aspirate. The management of thyroid nodules relies principally on a cytopathological diagnosis, which is reported using The Bethesda System for Reporting Thyroid Cytology (TBSRTC) [3]. Still, a considerable portion of thyroid nodules that undergo FNA are classified as indeterminate (20–25%), of which 10–60% of these are malignant [3].

Indeterminate thyroid nodules (ITNs) include Bethesda Class III, atypia of undetermined significance/follicular lesion of undetermined significance (AUS/FLUS), and Class IV, follicular neoplasm or suspicious for a follicular neoplasm (FN/SFN) [3]. Some literature includes Bethesda Class V, suspicious for malignancy, as well. Since these nodules are neither benign nor malignant on preoperative assessment, ITNs pose a challenge for clinical management [4]. Options for management include repeat FNA, molecular testing, patient monitoring, minimally-invasive ablative techniques, and hemithyroidectomy [3,4].

Advancement in thyroid tumorigenesis genetics has allowed the development of molecular testing to complement cytological diagnosis and improve the risk-based stratification of ITNs [5]. Molecular testing platforms are currently implicated to refine the preoperative diagnosis of ITNs by reporting a refined patient risk stratification, subsequently reducing the need for diagnostic surgery [5,6]. Two common molecular testing platforms include those developed by Afirma (San Francisco, CA, USA) and Interpace Diagnostics (Parsippany, NJ, USA), both of which have been described as good rule-out tests due to a high negative predictive value (NPV) [7]. The use of these genetic tests can assist clinicians and surgeons in patient risk stratification and management planning [7].

Interpace Diagnostics utilizes a genotyping panel, ThyGeNEXT, as well as a molecular test with a high NPV for thyroid cancer which utilizes a microRNA-based assay, ThyraMIR. MicroRNAs are small, noncoding ribonucleic acid molecules that play a role in regulating cellular function including cell cycle gene expression, proliferation, and survival [8]. Samples first undergo ThyGeNEXT evaluation, and if not determined to possess a strong/driver mutation, subsequently undergo further evaluation by ThyraMIR. “Strong” driver mutations include those with a strong probability of cancer, including the BRAF V600E mutation, TERT promoter mutations, and ALK mutations. The Interpace Diagnostics molecular testing platforms ThyGeNEXT and ThyraMIR possess a high NPV (95%) and are used clinically to rule-out thyroid cancer [9].

Veracyte Inc. (San Francisco, CA, USA) developed the Afirma Gene Expression Classifier (GEC), a microarray-based test with a proprietary algorithm that examines the mRNA expression of a panel of 167 genes. This molecular test was validated clinically in a blinded prospective multicenter trial including 265 thyroid nodules [10]. In 2018, Afirma developed and published its validation study with the improved Gene Sequencing Classifier (GSC) [11]. In addition to GSC evaluation, which measures RNA gene expression levels, Afirma developed the Xpression Atlas (XA) which can detect sequence variants, such as gene fusions, insertions, and deletions, as well as point mutations. The use of the latest Afirma technology demonstrated an impressive NPV of 96% [11]. The use of both the GSC and XA among ITNs provides valuable insight for clinical decision-making [12].

While several works have reported their experience with genetic testing platforms, there remain only a few works which investigate different genetic testing platforms from a single institution. For example, one work which analyzed three commercial testing platforms (Afirma, RosettaGX, and Interpace) reported only on a total of 70 nodules. The authors reported that all genetic testing platforms displayed high NPV [13]. Works which analyze only a single genetic testing platform are subject to inherent biases when compared to other single-institution works, considering the variation in cytopathologists’ assessments (i.e., intra-rater as well as inter-rater agreement), as well as the prevalence of thyroid cancer and mutations, which fundamentally alter the positive predictive value (PPV) and the NPV of these molecular tests. Considering these limitations, we performed a comparative analysis of molecular performance tests at a single institution. Our primary aim in this study was to assess the diagnostic performance of the latest Afirma (GSC + XA) and Interpace Diagnostics (ThyGeNEXT + ThyraMIR) platforms.

## 2. Material and Methods

### 2.1. Study Design

After obtaining institutional review board (IRB) approval from Tulane Medical Center, we reviewed all results of thyroid biopsies from patients with ITNs. All patients underwent FNA by a single, high-volume, fellowship-trained endocrine surgeon. Patients with ITNs according to a cytopathological analysis using Afirma and Interpace Diagnostics molecular testing data from November 2013 to November 2021 were included.

### 2.2. Ultrasound Assessment

All patients with thyroid nodules underwent comprehensive neck ultrasound evaluation. Ultrasound assessment was conducted using a 15-MHz linear transducer. Nodules were assessed by evaluating the three-dimensional diameter, composition, echogenicity, margins, echogenic foci, vascularity, and elastography of the nodules. Suspicious nodules underwent further evaluation with FNA.

### 2.3. Fine-Needle Aspiration

FNA biopsies were conducted utilizing a 10 mm 25-gauge needle with an uninterrupted real-time observation of the needle tip through each nodule via ultrasound guidance. The lower neck was prepared with povidone-iodine swabs and draped in a standard surgical fashion. A total of 2 mL of 1% lidocaine was injected as local anesthesia. Lidocaine cream (EMLA cream 5%) was used prior to injections. In the same FNA setting, samples were also collected for molecular testing in specified preservation tubes from Afirma or Interpace Diagnostics. Samples were subsequently sent for laboratory analysis to a cytopathologist at our institution. Consistent with most academic centers, further analysis by molecular testing was up to the discretion of the pathologist. At our practice, the vast majority of thyroid nodules which are not classified as benign (Bethesda I/III–VI) are sent for molecular testing. For the minority of patients who elect to undergo thyroidectomy, regardless of biopsy cytological analysis, genetic testing was not sent. The decision to send the biopsy specimen to Afirma or Interpace Diagnostics for evaluation by genetic testing was also up to the pathologist. The tendency to send nodules to Afirma for genetic testing can be explained by the fact that different providers at our institution each have their own preferences regarding their test of choice. This reflects the true nature of an endocrine practice. As a whole, however, pathologists did not have a preference for either genetic testing platform and sent specimens without any particular arrangement. The majority of the FNA were performed at our out-patient clinic, and a few were performed under general anesthesia in the operating room, per patient preference. FNA sites were matched to the respected nodules to the best of the pathologist’s knowledge.

### 2.4. Afirma Molecular Testing

Following evaluation by a cytopathologist, Bethesda III, IV, and V nodules were sent for further molecular testing. Implementation of XA use at our institution began in May 2018, when all patients began receiving the GSC and its complementary XA. FNA samples sent for genetic testing prior to this time-point were subject to either GEC or GSC testing alone. Specimen collection and shipping were in accord with Afirma’s recommendations. Afirma requires that additional aspirates be procured and submitted in special collection media and additionally requires that specimens be refrigerated and/or frozen prior to shipping [14].

### 2.5. Interpace Molecular Testing

Following evaluation by a cytopathologist, ITNs classified as Bethesda Class I, III, IV, and V nodules were sent for further molecular testing. Molecular testing was performed only for those patients with aspirates subject to the ThyGeNEXT panel, with its corresponding ThyraMIR panel. Patients receiving ThyGenX, which was introduced in 2014 and assesses for 100 genetic alterations across 8 different oncogenes [13], were excluded from this cohort, as there were only a few patients, and this technology is no longer in use. ThyGeNEXT was introduced in 2018 and includes additional markers. Specimen collection and shipping were in accord with Interpace Diagnostic’s recommendations [15]. Since Interpace Diagnostics also offers the ability to test nondiagnostic FNA specimens, Bethesda I specimen samples were also shipped.

All pathology reports were read by a single pathology department before being sent out for genetic testing by Afirma or Interpace Diagnostics. The results of molecular testing were identified using the Veracyte physician portal for Afirma results or the Interpace Diagnostics physician portal for Interpace Diagnostics results. Afirma molecular testing included the GEC, GSC, and XA. Interpace diagnostics molecular testing included ThyGeNEXT and ThyraMIR. Molecular testing results were then copied directly from the physician portals to a predesigned spreadsheet. Patients with insufficient genetic testing were removed. Electronic medical records were used to extract demographic information, nodule characteristics, and final pathology results associated with each thyroid nodule.

### 2.6. Cohorts

Considering that the primary aim of our study was to assess the diagnostic performance of the latest Afirma and Interpace Diagnostics platforms, we sub-grouped the study population by the molecular test that the patients received. Three cohorts were determined, including GSC + XA (the latest Afirma platform), ThyGeNEXT + ThyraMIR (a recent Interpace Diagnostics platform, only recently updated in 2022 with ThyraMIR v2 [16]), and GSC + GEC (Afirma testing platform prior to the introduction of the XA panel). The GSC has been proven superior to the GEC, including a higher benign-call rate (61.2% vs. 41.6%), and it was not further sub-stratified into individual cohorts, as this was not the primary concern of the study, and it has been reported on previously [11,17,18].

### 2.7. Statistical Analysis

IBM SPSS Statistics 27 was used for statistical analysis. A two-sided Chi-square test was used for analysis, and the *p*-values < 0.05 were set to be significant. Since clinician decision making and patient preference both play an important role in our practice, not all patients with ITNs underwent thyroidectomy. Therefore, we conducted an overall analysis including the whole study population using surgical pathology, if available, and conservative management follow-up decision making, if surgical pathology was not available. All patients not undergoing thyroidectomy were followed-up for at least 6 months to determine if there were any suspicious features or changes on ultrasound images. Typical criteria during the management of active surveillance patients, including nodule growth of more than 50% or suspicious lymph node involvement, were considered. Patient nodules not exhibiting these features were considered benign, while patients exhibiting these features were considered to have malignant nodules. While this reflects true clinical decision making, as well as the nature of an endocrine practice, we also conducted a sub-group analysis including only patients undergoing thyroidectomy who consequently have available surgical pathology to determine malignancy status. For Afirma, a benign genetic test was read as a negative test, while a suspicious test was read as a malignant test. For Interpace Diagnostics, low-risk was read as a benign test and an intermediate risk or high risk was read as a malignant test. The sensitivity, specificity, PPV, NPV, diagnostic odds ratio (DOR), positive likelihood ratio, negative likelihood ratio, and accuracy were determined. True positives were those nodules determined as suspicious by molecular testing and which were subsequently proven malignant by surgical pathology. True negatives were those nodules determined as benign by molecular testing and which were subsequently proven benign by surgical pathology. False positives were nodules determined as suspicious by molecular testing and which were subsequently proven benign by surgical pathology. False negatives were those nodules determined as benign by molecular testing and which were subsequently proven malignant by surgical pathology. Meta-disc version 4.0 was used for the analysis of the diagnostic accuracy measures and comparison between genetic testing platforms.

## 3. Results

### 3.1. Study Population

**Figure 1:**
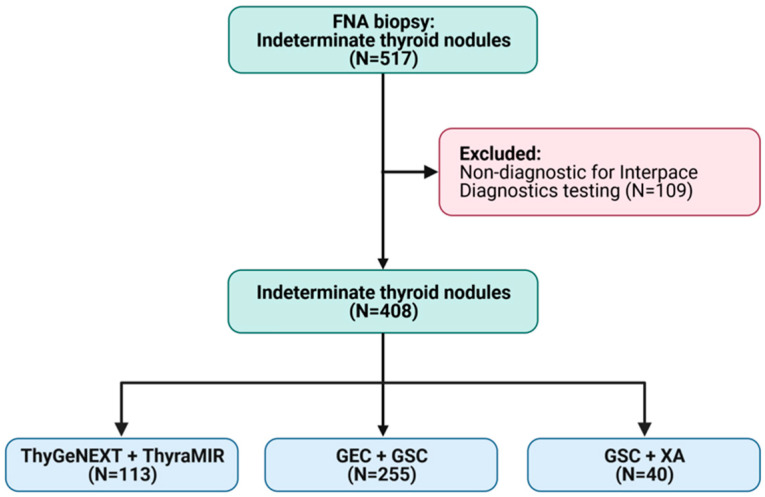
Breakdown of the study population. Figure made with BioRender (license: Tulane University).

A total of 517 FNA biopsies were performed. Of these, 109 were non-diagnostic and were sent for Interpace Diagnostics testing, which is beyond the scope of this study. This allowed for a total of 408 ITNs for our study population, including 113 in the ThyGeNEXT + ThyraMIR cohort, 255 in the GEC + GSC cohort, and 40 in the GSC + XA cohort (Figure 1).

The breakdown of the three cohorts is shown in Table 1. All cohorts were comprised predominately of Bethesda III nodules, including at least 85% or more. Bethesda V nodules comprised 8.0%, 11.4%, and 10% of the nodules in the ThyGeNEXT + ThyraMIR, GEC + GSC, and GSC + XA cohorts, respectively. Finally, Bethesda IV nodules comprised only 3.5% or less in each cohort.

**Figure 2:**
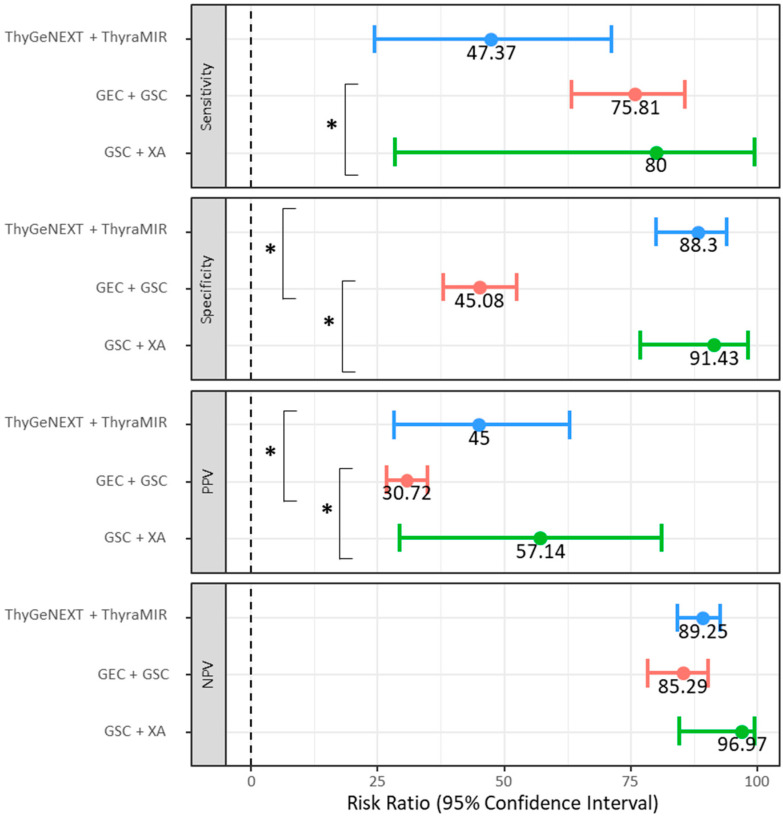
The sensitivity, specificity, positive predictive value, and negative predictive value in nodules undergoing the ThyGeNEXT + ThyraMIR, GEC + GSC, and GSC + XA molecular testing. PPV: positive predictive value; NPV: negative predictive value. * indicates *p* < 0.05.

A total of 408 ITNs were assessed. A total of 230 of these patients underwent surgery, accounting for 56.4% of the study population. All patients who did not undergo surgery were monitored for at least 6 months and deemed benign if there were no suspicious features or significant growth on their ultrasound images. Details of the diagnostic accuracy of nodules undergoing ThyGeNEXT + ThyraMIR, GEC + GSC, and GSC + XA molecular testing are depicted in Table 2. The sensitivity of the ThyGeNEXT + ThyraMIR, GEC + GSC, and GSC + XA cohorts was 47.37% (95%CI: 24.45–71.14%), 75.81% (95%CI: 63.26–85.78%), and 80.00% (95%CI: 28.36–99.49%), respectively (Figure 2). The sensitivity of GSC + XA was greater than that of GEC + GSC (*p* < 0.001), but not greater than that of ThyGeNEXT + ThyraMIR (*p* = 0.08). Nodules undergoing GEC + GSC had a lower specificity (45.08%, 95%CI: 37.92–52.39%) than those undergoing ThyGeNEXT + ThyraMIR (88.30%, 95%CI: 80.03–94.01%, *p* < 0.001) and GSC + XA (91.43%, 95%CI: 76.94–98.20%, *p* < 0.001). The PPV of ThyGeNEXT (*p* = 0.032) + ThyraMIR and GSC + XA (*p* < 0.001) was superior to that of GEC + GSC. The PPV was 45.00% (95%CI: 28.28–62.93%), 30.72% (95%CI: 26.83–34.90%), and 57.14% (95%CI: 29.32–81.08%), for the ThyGeNEXT + ThyraMIR, GEC + GSC, and GSC + XA cohorts, respectively. The NPV was similar and above 85% in all cohorts, suggesting an overall suitable rule-out test (*p* > 0.05). The DOR was not significantly different across cohorts (*p* > 0.05). Still, the DOR was relatively low at 2.57 (95%CI: 1.36–4.91) in the GEC + GSC cohort, high at 6.79 (95%CI: 2.26–20.37) in the ThyGeNEXT + ThyraMIR cohort, and very high at 42.67 (95%:CI 3.54–514.85) in nodules undergoing GSC + XA testing. The overall accuracy for nodules undergoing ThyGeNEXT + ThyraMIR and GSC + XA cohort testing was 81.42% (95%CI: 73.01–88.11%, *p* < 0.001) and 90.00% (95%CI: 76.34–97.21%, *p* < 0.001), respectively, and significantly greater than the accuracy for nodules undergoing GEC + GSC testing. The overall accuracy of nodules undergoing GEC + GSC was 52.55% (95%CI: 46.23–58.81%).

### 3.2. Sub-Group Analysis including Nodules Undergoing Surgery

**Figure 3:**
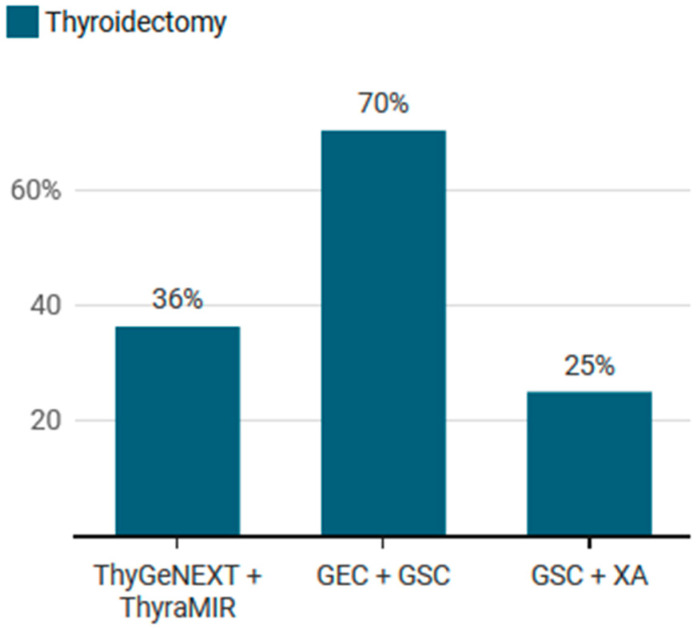
Frequency of nodules undergoing thyroidectomy sub-grouped by genetic testing platform.

A total of 230 patients underwent thyroidectomy and accordingly, had available surgical pathology. The surgical rate per cohort was significantly different (*p* < 0.001) by cohort. The rates of thyroidectomy in the ThyGeNEXT + ThyraMIR and GSC + XA cohorts were 36.3% and 25.0%, respectively (Figure 3). Conversely, 70.2% of the GEC + GSC cohort underwent thyroidectomy. The overall rate of malignancy in this sub-group was 36.52% (N = 84/230), with similar rates of malignancy between the cohorts (*p* > 0.05). Considering the low conversion rate to thyroidectomy in the ThyGeNEXT + ThyraMIR and GSC + XA cohorts, the count of nodules with surgical pathology was 41 and 10, respectively. The GEC + GSC cohort included a total of 179 nodules.

**Figure 4:**
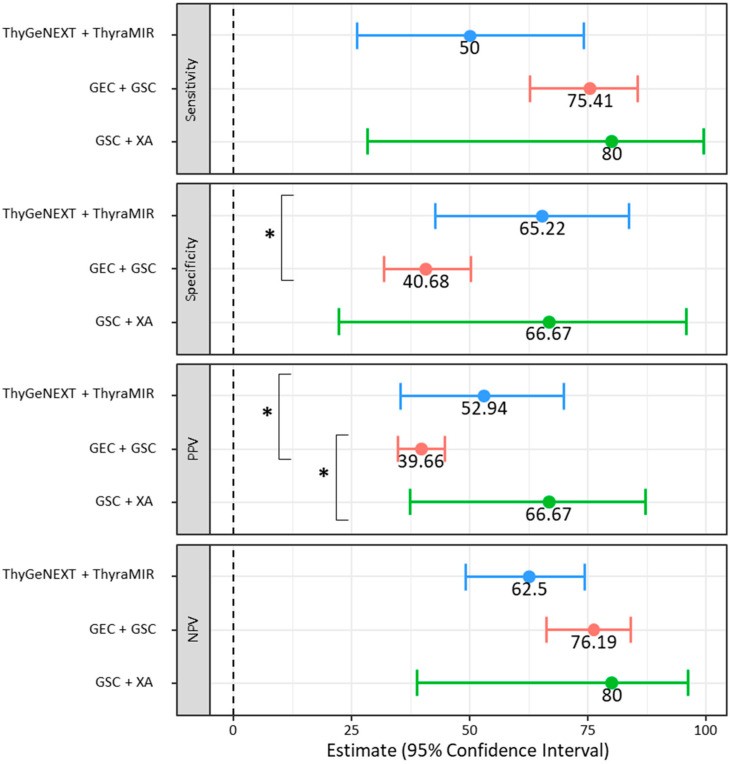
The sensitivity, specificity, positive predictive value, and negative predictive value with available surgical pathology in nodules undergoing the ThyGeNEXT + ThyraMIR, GEC + GSC, and GSC + XA molecular testing. PPV: positive predictive value; NPV: negative predictive value. * indicates *p* < 0.05.

For patients with available surgical pathology, the sensitivity of the three cohorts was similar, including ThyGeNEXT + ThyraMIR, GEC + GSC, and GSC + XA at 50.00% (95%CI: 26.02–73.98%), 75.41% (95%CI: 62.71–85.54%), and 80.00% (95%CI: 28.36–99.49%), respectively (*p* > 0.05) (Table 3). The specificity of ThyGeNEXT + ThyraMIR at 65.22% (95%CI: 42.73–83.62%) and GSC + XA at 66.67% (95%CI: 22.28–95.67%) were similar (*p* > 0.05) (Figure 4). The specificity for nodules undergoing ThyGeNEXT + ThyraMIR was significantly greater than nodules undergoing GEC + GSC testing (*p* = 0.026). The PPV was the highest in the ThyGeNEXT + ThyraMIR and GSC + XA cohorts at 52.94% (95%CI: 35.25–69.92%, *p* < 0.001) and 66.67% (95%CI: 37.28–87.06%, *p* = 0.015). The PPV for nodules undergoing GEC + GSC testing was 39.66% (95%CI: 34.82–44.70%). While there were no significant differences in NPV (*p* > 0.05), it was above 75% in the GEC + GSC and the GSC + XA cohorts at 76.19% (95%CI: 66.21–83.94%) and 80.00% (95%CI: 38.80–96.19%), respectively. The NPV in the ThyGeNEXT + ThyraMIR was 62.60% (95%CI: 49.02–74.28%). The DOR was similar across all cohorts (*p* > 0.05), including 1.88 (95%CI: 0.53–6.62) and 2.10 (95%CI: 1.06–4.19) in the ThyGeNEXT + ThyraMIR and GEC + GSC cohorts. The DOR remained high, but insignificant, in the GSC + XA cohort at 8.00 (95%CI: 0.50–127.90). The overall accuracy for the nodules undergoing ThyGeNEXT + ThyraMIR, GEC + GSC, and GSC + XA testing were 58.54% (95%CI: 42.11–73.68%), 52.51% (95%CI: 44.93–60.01%), and 72.73% (95%CI: 39.03–93.98%) (*p* > 0.05).

## 4. Discussion

Thyroid nodules are common and are being detected at increased rates over the past few decades [1,19]. Nodules represent a wide spectrum of pathology, including benign nodules, as well as high-risk carcinomas, which adversely affect patient survival. Standard first-line assessment of thyroid nodules includes preoperative ultrasound and, typically, FNA [2]. FNA cytological analysis heavily guides clinical judgment, although its diagnostic accuracy has not improved within recent decades [20]. Molecular testing enhances patient risk stratification. In our study, all three cohorts, including GSC + XA, ThyGeNEXT + ThyraMIR, and GSC + GEC, were effective in ruling-out thyroid cancer. Specifically, the use of ThyGeNEXT + ThyraMIR and GSC + XA decreased the surgical rate in patients with ITNs by more than 60%.

Surgery remains the mainstay for establishing a definitive histopathological diagnosis, especially among ITNs. Still, surgery carries the concerning risk of complications which impact patient quality of life, such as dysphonia and permanent hypothyroidism [21]. In a recent review, Schneider et al. reported that approximately 75% of ITNs undergoing thyroidectomy are benign on surgical pathology, suggesting unnecessary surgical intervention [22]. In their 60-patient ITN cohort study, Balentine et al. reported only a 6% rate of malignancy in surgical pathology [23]. Importantly, the authors noted that 47% of patients develop hypothyroidism following thyroidectomy [23]. Accordingly, the importance of accurate pre-operative risk stratification by molecular testing cannot be understated.

Molecular tests are increasingly utilized as ancillary tools to avoid diagnostic surgery as an approach for cytologically ITNs. For example, Angell et al. reported on 600 nodules and found a surgery rate of 45.4% (221/486) and 28.1% (32/114) in the GEC and GSC cohorts, respectively [17]. While our study stratified cohorts slightly differently, we also found that the newer Afirma technology (GSC + XA) significantly decreased the rate of surgery in these patients. The authors reported a PPV of 33.9% (75/221) with the GEC and 50% (16/32) with the use of the GSC [17]. The PPV using ThyGeNEXT + ThyraMIR is reported to be 52% [9]. In our study population, we reported similar PPVs of 45% and 57.14% using ThyGeNEXT + ThyraMIR and GSC + XA, respectively. These values understandably increased to 52.94% and 66.67%, respectively, when sub-grouped by patients who underwent surgery. Our work suggests a similar PPV to that of a 2022 meta-analysis including 13 studies evaluating the diagnostic performance of the Afirma GSC, reporting a PPV of 64.9% [24]. The NPV of ThyGeNEXT + ThyraMIR and Afirma GSC are 95% and 96%, respectively [9]. We found similar NPVs, with 89.25% and 96.97%, in our study population which decreased to 62.50% and 80%, when including only patients who underwent surgery. The increase in PPV and decrease in NPV can be explained considering that patients with higher risks of malignancy (i.e., suspicious sonographic features or increases in nodule size) were more likely to undergo thyroidectomy. The increase in PPV and decrease in NPV can be explained by considering two factors. The first is that the prevalence of malignancy in our patient population is higher than that of the general population. We previously published these findings [25,26], reporting a prevalence of malignancy in ITNs which is at the upper-end and/or higher than ranges reported from other institutions (10–19% at Mount Sinai Beth Israel, 30–38% at Memorial Sloan Kettering Cancer Center) [27]. The second, which is related to the first, is that patients with higher risks of malignancy (i.e., suspicious sonographic features or increases in nodule size) were more likely to undergo thyroidectomy. Importantly, this allowed a malignancy rate of 36.52% in patients with ITNs, much higher than the 6% reported by Balentine et al. [23], demonstrating the importance regarding the selection of patients with suspicious nodules, and the obviation of surgery in patients who may not have required it. With NPVs of 89% and higher in nodules undergoing genetic testing with either of these platforms, our work corroborates the current literature suggesting both ThyGeNEXT + ThyraMIR and GSC + XA to be effective rule-out genetic tests. Specifically, the NPV of GSC + XA (96.97%) tended to be higher than that of ThyGeNEXT + ThyraMIR (89.25%) genetic testing. It is the opinion of the authors that genetic testing should be a first-line option for patients with ITNs.

FNA is widely performed to allow for a minimally-invasive mechanism of determining nodular cytology. A recent 2022 meta-analysis including 16,697 patients (36 studies) found that the sensitivity and specificity of FNA was 85.6% (95%CI:, 79.9–89.5%) and 71.4% (95%CI: 61.1–79.8%), respectively [20]. While the diagnostic accuracy of FNA is high, the authors noted that the accuracy of FNA has not increased over time, including studies published as far back as 1982 [20]. This primarily highlights the importance of accurate genetic testing, but also the importance of clinical decision making. For example, a patient with a Bethesda III nodule which is only 1 cm and without suspicious sonographic features may be suitable for non-surgical management and close patient monitoring. Accordingly, our population-level analysis determined by conservative management follow-up or final surgical pathology, if available, possesses merit, as it reflects a true endocrine practice.

Several reviews have discussed the strengths and weaknesses of each of these genetic testing platforms beyond their diagnostic accuracy [7,9]. While details of these discussions are beyond the scope of this work, including the number of genes assessed by gene expression analysis or the number of variants assessed, several factors are worth mentioning. Foremost, there are financial considerations regarding genetic testing. Since thyroidectomy is costly (considering operating room time, staffing fees, equipment, time away from work for the patient, complications, etc.), the use of molecular testing has been suggested to be a cost-effective management option [28,29], although other works still suggest the opposite [30,31]. Determining which genetic testing platform is the most cost-effective is complicated, given that some patients may require additional testing, which is purchased separately. For example, the cost of BRAF mutation genetic testing alone is USD 475 USD, while the MTC genetic testing alone is 975 USD from Afirma [32]. Additionally, different works suggest different rates of surgery following genetic testing, further blurring a transparent cost-effective analysis. In our study, the use of either ThyGeNEXT + ThyraMIR or GSC + XA decreased the rate of thyroidectomy by more than 60%. In 2019, one study found that the use of GSC decreased the rate of thyroidectomy by 45% compared with that for GEC alone [18]. Specifically, our work found that Afirma’s GSC + XA decreased the rate of surgery to 25%, which was more than that of Interpace Diagnostic’s ThyGeNEXT + ThyraMIR (which decreased the rate to 36.3%). Recently, Sunoco et al. incorporated sonographic features in their cost-effective analysis and determined that molecular testing was only cost-effective for ITNs with either an intermediate or a low suspicion of malignancy [33]. Nicholson et al. also reported that molecular testing, specifically either GSC or ThyroSeq version 3, is considerably more cost-effective than diagnostic lobectomy for patients with ITNs who lack other indications for thyroidectomy [29]. Future works incorporating nodule sonographic features, similar to the work of Zanocco et al., as well as considering the different commercially available genetic tests, are warranted to better elucidate whether genetic testing is more cost-effective than diagnostic lobectomy and to facilitate the comparison of molecular platforms.

One advantage of the utilization of the Interpace Diagnostics genetic testing platform is their ease of specimen collection. Interpace Diagnostics does not require a dedicated FNA, but rather samples can be collected as cells on a direct smear or as cells/wash-out collected from an FNA pass [11]. Conversely, Afirma requires a dedicated two-pass FNA [9]. Therefore, the use of the Interpace Diagnostics platform may be more appropriate in patients who experience considerable discomfort during FNA biopsy.

There are limitations to our study. Foremost, the retrospective study design of the work is subject to inherent biases. Furthermore, the authors acknowledge that the population-level analysis including patients monitored for at least 6 months, but without surgical pathology, may artificially inflate the true negative rate. In consequence, the sub-group analysis was performed including only patients with surgical pathology. Another minor limitation of this study was our obviation of patients analyzed by the newest Interpace Diagnostics risk classifier, ThyraMIR v2 [16]. This technology was only recently introduced in 2022 and aims to further improve ITN risk stratification. One strength of this work is its investigation of different genetic testing platforms within the same institution.

## 5. Conclusions

ThyGeNEXT + ThyraMIR and GSC + XA are genetic tests with effective rule-out abilities. The use of these genetic testing platforms, specifically ThyGeNEXT + ThyraMIR and GSC + XA, decreased the surgical rate in patients with ITNs by more than 60%.

## Figures and Tables

**Table 1:** Breakdown of molecular testing by the Bethesda classification.

Bethesda Classification	ThyGeNEXT + ThyraMIR (N = 113)	GEC + GSC (N = 255)	GSC + XA (N = 40)
Bethesda III	100 (88.5)	221 (86.7)	35 (87.5)
Bethesda IV	4 (3.5)	5 (2.0)	1 (2.5)
Bethesda V	9 (8.0)	29 (11.4)	4 (10)

Data is reported as count and percentage of cohort. Comparison of molecular tests.

**Table 2:** Diagnostic accuracy of nodules undergoing the ThyGeNEXT + ThyraMIR, GEC + GSC, and GSC + XA molecular testing.

	ThyGeNEXT + ThyraMIR (N = 113)		GEC + GSC (N = 255)		GSC + XA (N = 40)	
	Value	95%CI	Value	95%CI	Value	95%CI
Sensitivity	47.37	24.45–71.14	75.81	63.26–85.78	80.00	28.36–99.49
Specificity	88.30	80.03–94.01	45.08	37.92–52.39	91.43	76.94–98.20
Positive LR	4.05	1.95–8.40	1.38	1.14–1.67	9.33	2.90–29.99
Negative LR	0.60	0.39–0.92	0.54	0.34–0.86	0.22	0.04–1.27
PPV	45.00	28.28–62.93	30.72	26.83–34.90	57.14	29.32–81.08
NPV	89.25	84.34–92.75	85.29	78.42–90.25	96.97	84.68–99.46
DOR	6.79	2.26–20.37	2.57	1.35–4.91	42.67	3.54–514.85
Accuracy	81.42	73.01–88.11	52.55	46.23–58.81	90.00	76.34–97.21

Data are presented as a value and 95% confidence interval (lower limit—upper limit). A gold highlight indicates a superior genetic testing platform for that diagnostic parameter. LR: likelihood ratio; PPV: positive predictive value; NPV: negative predictive value; DOR: diagnostic odds ratio; CI: confidence interval.

**Table 3:** Diagnostic accuracy of nodules with available surgical pathology undergoing the ThyGeNEXT + ThyraMIR, GEC + GSC, and GSC + XA molecular testing.

	ThyGeNEXT + ThyraMIR (N = 41)		GEC + GSC (N = 179)		GSC + XA (N = 10)	
	Value	95%CI	Value	95%CI	Value	95%CI
Sensitivity	50.00	26.02–73.98	75.41	62.71–85.54	80.00	28.36–99.49
Specificity	65.22	42.73–83.62	40.68	31.73–50.11	66.67	22.28–95.67
Positive LR	1.44	0.70–2.97	1.27	1.03–1.56	2.40	0.71–8.08
Negative LR	0.77	0.44–1.33	0.60	0.37–0.99	0.30	0.05–1.89
PPV	52.94	35.25–69.92	39.66	34.82–44.70	66.67	37.28–87.06
NPV	62.50	49.02–74.28	76.19	66.21–83.94	80.00	38.80–96.19
DOR	1.88	0.53–6.62	2.10	1.06–4.19	8.00	0.50–127.90
Accuracy	58.54	42.11–73.68	52.51	44.93–60.01	72.73	39.03–93.98

Data are present for value and 95% confidence interval (lower limit—upper limit). A gold highlight indicates a superior genetic testing platform for that diagnostic parameter. LR: likelihood ratio; PPV: positive predictive value; NPV: negative predictive value; DOR: diagnostic odds ratio; CI: confidence interval.

## Data Availability

The data is contained within the article.

## References

[1] Guth S., Theune U., Aberle J., Galach A., Bamberger C. (2009). Very High Prevalence of Thyroid Nodules Detected by High Frequency (13 MHz) Ultrasound Examination. Eur. J. Clin. Investig..

[2] Haugen B.R., Alexander E.K., Bible K.C., Doherty G.M., Mandel S.J., Nikiforov Y.E., Pacini F., Randolph G.W., Sawka A.M., Schlumberger M. (2016). 2015 American Thyroid Association Management Guidelines for Adult Patients with Thyroid Nodules and Differentiated Thyroid Cancer: The American Thyroid Association Guidelines Task Force on Thyroid Nodules and Differentiated Thyroid Cancer. Thyroid.

[3] Cibas E.S., Ali S.Z. (2017). The 2017 Bethesda System for Reporting Thyroid Cytopathology. Thyroid.

[4] Durante C., Grani G., Lamartina L., Filetti S., Mandel S.J., Cooper D.S. (2018). The Diagnosis and Management of Thyroid Nodules: A Review. JAMA.

[5] Vargas-Salas S., Martínez J.R., Urra S., Domínguez J.M., Mena N., Uslar T., Lagos M., Henríquez M., González H.E. (2018). Genetic Testing for Indeterminate Thyroid Cytology: Review and Meta-Analysis. Endocr. Relat. Cancer.

[6] Silaghi C.A., Lozovanu V., Georgescu C.E., Georgescu R.D., Susman S., Năsui B.A., Dobrean A., Silaghi H. (2021). Thyroseq v3, Afirma GSC, and MicroRNA Panels versus Previous Molecular Tests in the Preoperative Diagnosis of Indeterminate Thyroid Nodules: A Systematic Review and Meta-Analysis. Front. Endocrinol..

[7] Nishino M. (2016). Molecular Cytopathology for Thyroid Nodules: A Review of Methodology and Test Performance. Cancer Cytopathol..

[8] Lithwick-Yanai G., Dromi N., Shtabsky A., Morgenstern S., Strenov Y., Feinmesser M., Kravtsov V., Leon M.E., Hajdúch M., Ali S.Z. (2017). Multicentre Validation of a MicroRNA-Based Assay for Diagnosing Indeterminate Thyroid Nodules Utilising Fine Needle Aspirate Smears. J. Clin. Pathol..

[9] Nishino M., Bellevicine C., Baloch Z. (2021). Molecular Tests for Risk-Stratifying Cytologically Indeterminate Thyroid Nodules: An Overview of Commercially Available Testing Platforms in the United States. J. Mol. Pathol..

[10] Alexander E.K., Kennedy G.C., Baloch Z.W., Cibas E.S., Chudova D., Diggans J., Friedman L., Kloos R.T., LiVolsi V.A., Mandel S.J. (2012). Preoperative Diagnosis of Benign Thyroid Nodules with Indeterminate Cytology. N. Engl. J. Med..

[11] Patel K.N., Angell T.E., Babiarz J., Barth N.M., Blevins T., Duh Q.-Y., Ghossein R.A., Harrell R.M., Huang J., Kennedy G.C. (2018). Performance of a Genomic Sequencing Classifier for the Preoperative Diagnosis of Cytologically Indeterminate Thyroid Nodules. JAMA Surg..

[12] Angell T.E., Wirth L.J., Cabanillas M.E., Shindo M.L., Cibas E.S., Babiarz J.E., Hao Y., Kim S.Y., Walsh P.S., Huang J. (2019). Analytical and Clinical Validation of Expressed Variants and Fusions from the Whole Transcriptome of Thyroid FNA Samples. Front. Endocrinol..

[13] Partyka K.L., Trevino K., Randolph M.L., Cramer H., Wu H.H. (2019). Risk of Malignancy and Neoplasia Predicted by Three Molecular Testing Platforms in Indeterminate Thyroid Nodules on Fine-needle Aspiration. Diagn. Cytopathol..

[14] VanderLaan P.A. (2015). Molecular Markers: Implications for Cytopathology and Specimen Collection. Cancer Cytopathol..

[15] Benjamin H., Schnitzer-Perlman T., Shtabsky A., Vanden Bussche C.J., Ali S.Z., Kolar Z., Pagni F., Bar D., Meiri E., Rosetta Genomics Group (2016). Analytical Validity of a MicroRNA-based Assay for Diagnosing Indeterminate Thyroid FNA Smears from Routinely Prepared Cytology Slides. Cancer Cytopathol..

[16] Finkelstein S.D., Sistrunk J.W., Malchoff C., Thompson D.V., Kumar G., Timmaraju V.A., Repko B., Mireskandari A., Evoy-Goodman L.A., Massoll N.A. (2022). A Retrospective Evaluation of the Diagnostic Performance of an Interdependent Pairwise MicroRNA Expression Analysis with a Mutation Panel in Indeterminate Thyroid Nodules. Thyroid.

[17] Angell T.E., Heller H.T., Cibas E.S., Barletta J.A., Kim M.I., Krane J.F., Marqusee E. (2019). Independent Comparison of the Afirma Genomic Sequencing Classifier and Gene Expression Classifier for Cytologically Indeterminate Thyroid Nodules. Thyroid.

[18] Harrell R.M., Eyerly-Webb S.A., Golding A.C., Edwards C.M., Bimston D.N. (2019). Statistical Comparison of Afirma GSC and Afirma GEC Outcomes in a Community Endocrine Surgical Practice: Early Findings. Endocr. Pract..

[19] Davies L., Ouellette M., Hunter M., Welch H.G. (2010). The Increasing Incidence of Small Thyroid Cancers: Where Are the Cases Coming From?. Laryngoscope.

[20] Hsiao V., Massoud E., Jensen C., Zhang Y., Hanlon B.M., Hitchcock M., Arroyo N., Chiu A.S., Fernandes-Taylor S., Alagoz O. (2022). Diagnostic Accuracy of Fine-Needle Biopsy in the Detection of Thyroid Malignancy: A Systematic Review and Meta-Analysis. JAMA Surg..

[21] Bergenfelz A., Jansson S., Kristoffersson A., Mårtensson H., Reihnér E., Wallin G., Lausen I. (2008). Complications to Thyroid Surgery: Results as Reported in a Database from a Multicenter Audit Comprising 3660 Patients. Langenbecks Arch. Surg..

[22] Schneider D.F., Stafford L.M.C., Brys N., Greenberg C.C., Balentine C.J., Elfenbein D.M., Pitt S.C. (2017). Gauging the Extent of Thyroidectomy for Indeterminate Thyroid Nodules: An Oncologic Perspective. Endocr. Pract..

[23] Balentine C.J., Domingo R.P., Patel R., Laucirica R., Suliburk J.W. (2013). Thyroid Lobectomy for Indeterminate FNA: Not without Consequences. J. Surg. Res..

[24] Nasr C.E., Andrioli M., Endo M., Harrell R.M., Livhits M.J., Osakwe I., Polavarapu P., Siperstein A., Wei S., Zheng X. (2022). Real-World Performance of the Afirma Genomic Sequencing Classifier (GSC)—A Meta-Analysis. J. Clin. Endocrinol. Metab..

[25] Al-Qurayshi Z., Deniwar A., Thethi T., Mallik T., Srivastav S., Murad F., Bhatia P., Moroz K., Sholl A.B., Kandil E. (2017). Association of Malignancy Prevalence with Test Properties and Performance of the Gene Expression Classifier in Indeterminate Thyroid Nodules. JAMA Otolaryngol. Neck Surg..

[26] Al-Qurayshi Z., Sholl A.B., Kandil E. (2017). Exclusion of Eligible Indeterminate Thyroid Nodules in Estimates of Negative Predictive Value for the Gene Expression Classifier—Reply. JAMA Otolaryngol. Neck Surg..

[27] Marti J.L., Avadhani V., Donatelli L.A., Niyogi S., Wang B., Wong R.J., Shaha A.R., Ghossein R.A., Lin O., Morris L.G. (2015). Wide Inter-Institutional Variation in Performance of a Molecular Classifier for Indeterminate Thyroid Nodules. Ann. Surg. Oncol..

[28] Kuo J.H., McManus C., Graves C.E., Madani A., Khokhar M.T., Huang B., Lee J.A. (2019). Updates in the Management of Thyroid Nodules. Curr. Probl. Surg..

[29] Nicholson K.J., Roberts M.S., McCoy K.L., Carty S.E., Yip L. (2019). Molecular Testing versus Diagnostic Lobectomy in Bethesda III/IV Thyroid Nodules: A Cost-Effectiveness Analysis. Thyroid.

[30] Najafzadeh M., Marra C.A., Lynd L.D., Wiseman S.M. (2012). Cost-Effectiveness of Using a Molecular Diagnostic Test to Improve Preoperative Diagnosis of Thyroid Cancer. Value Health.

[31] Eszlinger M., Lau L., Ghaznavi S., Symonds C., Chandarana S.P., Khalil M., Paschke R. (2017). Molecular Profiling of Thyroid Nodule Fine-Needle Aspiration Cytology. Nat. Rev. Endocrinol..

[32] Sahli Z.T., Smith P.W., Umbricht C.B., Zeiger M.A. (2018). Preoperative Molecular Markers in Thyroid Nodules. Front. Endocrinol..

[33] Zanocco K.A., Wu J.X., Yeh M.W. (2017). Parathyroidectomy for Asymptomatic Primary Hyperparathyroidism: A Revised Cost-Effectiveness Analysis Incorporating Fracture Risk Reduction. Surgery.

